# 

**Published:** 1876-09

**Authors:** 


					﻿Vol. 23.
SEPTEMBER, 1876.
No. 9.
TWENTY-THIRD YEAR.
HALL’S
Journal of Health.
PUBLISHED AT 137 EIGHTH STREET, NEW YORK,
Four Doors East of 756 Broadway.
Copyright 1876, by E. H. Gibbs.
'	AGENTS FOR THE TRADE:
AMERICAN NEWS COMPANY, 121 NASSAU STREET.
NEW YORK NEWS COMPANY, 18 BEEKMAN STREET.
Terms, $2 a Year.
SINGLE NUMBER, 20 CENTS.
John Kent. Ptbirnr. 11 Frankfort Sr.. New York.
				

